# Smoking cessation at diagnosis and cancer survival: systematic review and meta-analysis

**DOI:** 10.1093/eurpub/ckac129.555

**Published:** 2022-10-25

**Authors:** M Del Riccio, G Bonaccorsi, C Lorini, V Vettori, S Gandini, S Caini

**Affiliations:** 1 Department of Health Sciences, University of Florence, Florence, Italy; 2 Department of Experimental Oncology, European Institute of Oncology, IRCCS, Milan, Italy; 3 ISPRO, Florence, Italy

## Abstract

Stopping smoking can considerably cut one's risk of developing cancer compared to continued smoking (i.e. up to 50% after 5 years for esophageal cancer and after 10 years for lung cancer). Much less is known about whether quitting smoking may bring a survival advantage to people who are active smokers at the time of cancer diagnosis. We conducted a systematic review and meta-analysis of the studies that examined the prognostic effect of quitting smoking at or around diagnosis among cancer patients. We searched MEDLINE and EMBASE for articles published until 30th March 2022 that reported the impact of quitting smoking at or around diagnosis on cancer patients’ survival (any type). Separately for each cancer site, study-specific data were pooled into summary relative risk (SRR) and corresponding 95% confidence intervals (CI) using random effect meta-analysis models, investigating sources of heterogeneity and bias. Forty-three articles were included, including 20 for lung cancer (LC), 16 for head and neck cancer (HNC), and less than 10 for bladder, breast, gastrointestinal tract, and other sites. Quitting smoking at or around diagnosis was associated with longer overall survival (SRR 0.71, 95% CI 0.64-0.80) in LC patients (consistently for non-small cell and small cell LC) as well as HNC patients (SRR 0.80, 95% CI 0.70-0.91). No significant publication bias was found. For the other body sites, the studies were limited in number, which prevented meta-analyses, but results were generally consistent with a beneficial effect of smoking cessation on survival. Quitting smoking at or around diagnosis is associated with a significantly improved overall survival of smokers diagnosed with LC and HNC and shows beneficial effects in patients with other cancers. Physicians should offer smoking cessation counselling to smokers who start diagnostic workup for suspected cancer, and smoking cessation strategies should arguably become part of standard multidisciplinary oncological care.

**Key messages:**

• Smoking cessation at or around diagnosis is associated with a significantly improved overall survival of smokers diagnosed with different types of cancer.

• Smoking cessation strategies (and counselling) should become part of standard multidisciplinary oncological care.

